# Unraveling Reticulate Evolution in *Opuntia* (Cactaceae) From Southern Mexico

**DOI:** 10.3389/fpls.2020.606809

**Published:** 2021-01-13

**Authors:** Xochitl Granados-Aguilar, Carolina Granados Mendoza, Cristian Rafael Cervantes, José Rubén Montes, Salvador Arias

**Affiliations:** ^1^Posgrado en Ciencias Biológicas, Instituto de Biología, Universidad Nacional Autónoma de México, Mexico City, Mexico; ^2^Jardín Botánico, Instituto de Biología, Universidad Nacional Autónoma de México, Mexico City, Mexico; ^3^Departamento de Botánica, Instituto de Biología, Universidad Nacional Autónoma de México, Mexico City, Mexico

**Keywords:** Cactaceae, *Opuntia*, reticulate evolution, Tehuacán-Cuicatlán Valley, hybridization, phylogenetic networks, nuclear markers, introgression

## Abstract

The process of hybridization occurs in approximately 40% of vascular plants, and this exchange of genetic material between non-conspecific individuals occurs unequally among plant lineages, being more frequent in certain groups such as *Opuntia* (Cactaceae). This genus is known for multiple taxonomic controversies due to widespread polyploidy and probable hybrid origin of several of its species. Southern Mexico species of this genus have been poorly studied despite their great diversity in regions such as the Tehuacán-Cuicatlán Valley which contains around 12% of recognized Mexico’s native *Opuntia* species. In this work, we focus on testing the hybrid status of two putative hybrids from this region, *Opuntia tehuacana* and *Opuntia pilifera*, and estimate if hybridization occurs among sampled southern opuntias using two newly identified nuclear intron markers to construct phylogenetic networks with HyDe and Dsuite and perform invariant analysis under the coalescent model with HyDe and Dsuite. For the test of hybrid origin in *O. tehuacana*, our results could not recover hybridization as proposed in the literature, but we found introgression into *O. tehuacana* individuals involving *O. decumbens* and *O. huajuapensis*. Regarding *O. pilifera*, we identified *O. decumbens* as probable parental species, supported by our analysis, which sustains the previous hybridization hypothesis between *Nopalea* and *Basilares* clades. Finally, we suggest new hybridization and introgression cases among southern Mexican species involving *O. tehuantepecana* and *O. depressa* as parental species of *O. velutina* and *O. decumbens*.

## Introduction

Reticulate evolution is a speciation pattern from which new species arise from hybridization and successive reproductive isolation (Soltis, 2013). In plants, hybridization takes place in approximately 40% of vascular species, can lead sometimes to speciation (Whitney et al., 2010), and is considered a major evolutive force (Soltis and Soltis, 2009; Soltis, 2013). Hybridization is the exchange of genetic material between individuals belonging to diagnostically distinct groups based on one or more heritable characters (Riesenberg and Wendel, 1993). This exchange of genetic material can lead to discordant phylogenetic trees, particularly in groups where hybridization is predominant (Arnold, 1997). Phylogenetic networks improve the representation of hybridization compared to phylogenetic trees (Zhu et al., 2018) because they extend trees with horizontal edges to better model such reticulation events (Elworth et al., 2019) and have been used to better depict the evolutive history of several lineages across vascular plants, including *Pinus* (Gernandt et al., 2018), *Viola* (Marcussen et al., 2011), *Fragaria* (Kamneva et al., 2017), and *Lachemilla* (Morales-Briones et al., 2018).

The genus *Opuntia* is known for its multiple taxonomic controversies resulting from wide morphological variability, potentially due to widespread polyploidy, hybridization between closely related species and, in some cases, homoplasy of selected morphological characters (Majure et al., 2012b). Among *Opuntia* species, natural hybrids are common, and hybridization has been regarded as having an impact on evolution (Pinkava, 2002; Reyes-Agüero et al., 2006; Majure et al., 2012a). Natural hybridization in this group is thought to be facilitated by the absence of reproductive barriers between closely related species, whose offspring are maintained by vegetative propagation and the perennial habit characteristics of this group (Pinkava, 2002; Majure and Puente, 2014). From the 180 recognized *Opuntia* species 93 are distributed in Mexico (Anderson, 2001; Hunt et al., 2006; Majure et al., 2012a). *Opuntia* species from southern Mexico have been poorly studied despite the great diversity found in regions such as the Tehuacán-Cuicatlán Valley, which has 12% (15 species) of the *Opuntia* species diversity of Mexico (Arias et al., 2012). Analyzing evolutive processes such as hybridization in a selected group could help us to better understand the mechanisms that have generated high diversity in this region.

**FIGURE 1:**
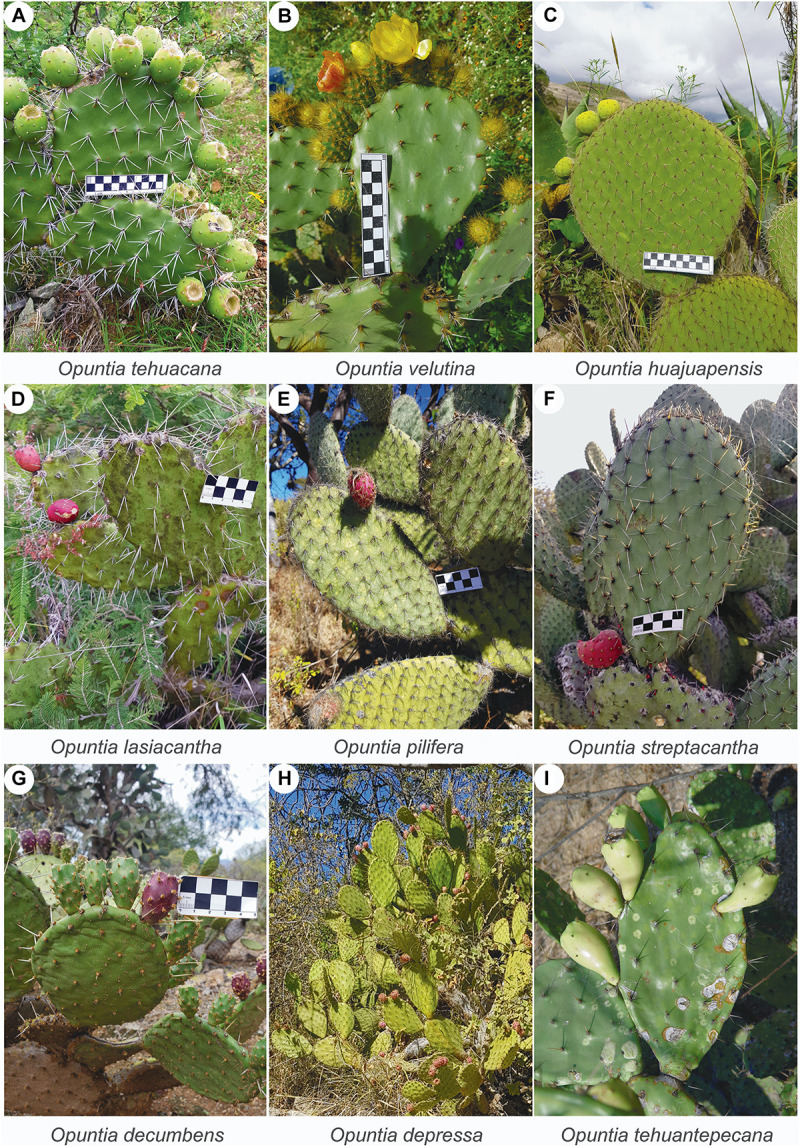
**(A–I)** Images of the nine *Opuntia* species analyzed on this work. **(C,E)** Species *O. huajuapensis* and *O. pilifera* putative parentals of *O. tehuacana*, as proposed by Arias et al. (2012). Photos by X. Granados and S. Arias.

Multiple species of probable hybrid origin have been suggested based on the incongruence of phylogenetic relationships depending on the inheritance patterns (i.e., uniparental vs. biparental; Majure et al., 2012a); however, these hypotheses have not been tested under coalescence reticulation inferences. One such case is *Opuntia pilifera* (Figure 1E), a prickly pear with edible fruits and wide trait variation, which was proposed as a hybrid between *Opuntia* species from *Nopalea* and *Basilares* clades (Majure et al., 2012a), without further inference of its potential parental species. Other species have been proposed to have a hybrid origin based only on morphological observation and without being examined in a phylogenetic context. One such case is *Opuntia tehuacana* (Figure 1A) which has been proposed as hybrid based on intermediate morphological traits between the sympatric species *O. pilifera* (Figure 1E) and *O. huajuapensis* (Figure 1C) from the Tehuacán-Cuicatlán Valley (Arias et al., 2012), but this hypothesis has never been formally tested.

Phylogenetic networks are an important approach to distinguish hybridization events in evolutive relationships that cannot be explained in a phylogenetic tree. Most of the phylogenetic analysis developed during the second half of the twentieth century focused on solving dichotomous relationships and were constrained by the low computational power of that time, leaving aside processes such as hybridization or incomplete lineage sorting (ILS) (Arnold, 1997; Elworth et al., 2019). Over the past 10 years improvement on computational power and implementation of statistical methods in phylogenetic software to detect reticulations and ILS led to new approaches based on parsimony, maximum likelihood and Bayesian inference. Under parsimony the inference of phylogenetic networks can be made from heuristic searches within a set of gene-tree topologies (Yu et al., 2013). The maximum likelihood approach is based on the multispecies network coalescent (MSNC) and maximizes the network likelihood (Yu et al., 2012). Bayesian inference, also uses the MSNC but includes a Markov chain Monte Carlo (MCMC) to sample posterior distribution on networks (Zhang et al., 2018). Examples of programs that can infer phylogenetic networks using these three approaches are SplitsTree4 (Huson and Bryant, 2005), BEAST 2 (Zhang et al., 2018), StarBEAST2 (Ogilvie et al., 2017), PhyloNetworks (Solís-Lemus et al., 2017), and PhyloNet (Wen et al., 2018). Among these, PhyloNet is one of the most commonly used (Copetti et al., 2017; Kamneva et al., 2017; Gernandt et al., 2018), since it allows the analysis of data from multiple loci through parsimony, maximum likelihood, pseudolikelihood, and Bayesian inference (Wen et al., 2018). All these software use coalescence theory to model the past of an allele using a stochastic process in order to find its most recent common ancestor. The mathematical approach in this theory also allows the estimation of the nucleotide mutation rate, and this methodology it is not affected by processes such as recombination (Rosenberg and Nordborg, 2002). Other approaches under the coalescent model to explore if hybridization occurs between certain taxa are HyDe, which uses phylogenetic invariants from site pattern probabilities to know the parental species of a putative hybrid (Blischak et al., 2018) and Dsuite, which test the correlations of alleles across populations using the Patterson’s *D* statistics (Malinsky et al., 2020).

Hybridization is a common process among *Opuntia* species and it study under phylogenetic networks can help to better understand the relationships of southern Mexico opuntias. This study aims to test the status of two putative hybrids from the Tehuacán-Cuicatlán Valley, *O. tehuacana* (Figure 1A) and *O. pilifera* (Figure 1E), which were previously proposed by Arias et al. (2012) and Majure et al. (2012a), respectively, and to estimate if hybridization occurs among sympatric *Opuntia* species.

## Materials and Methods

### Taxon Sampling

**FIGURE 2:**
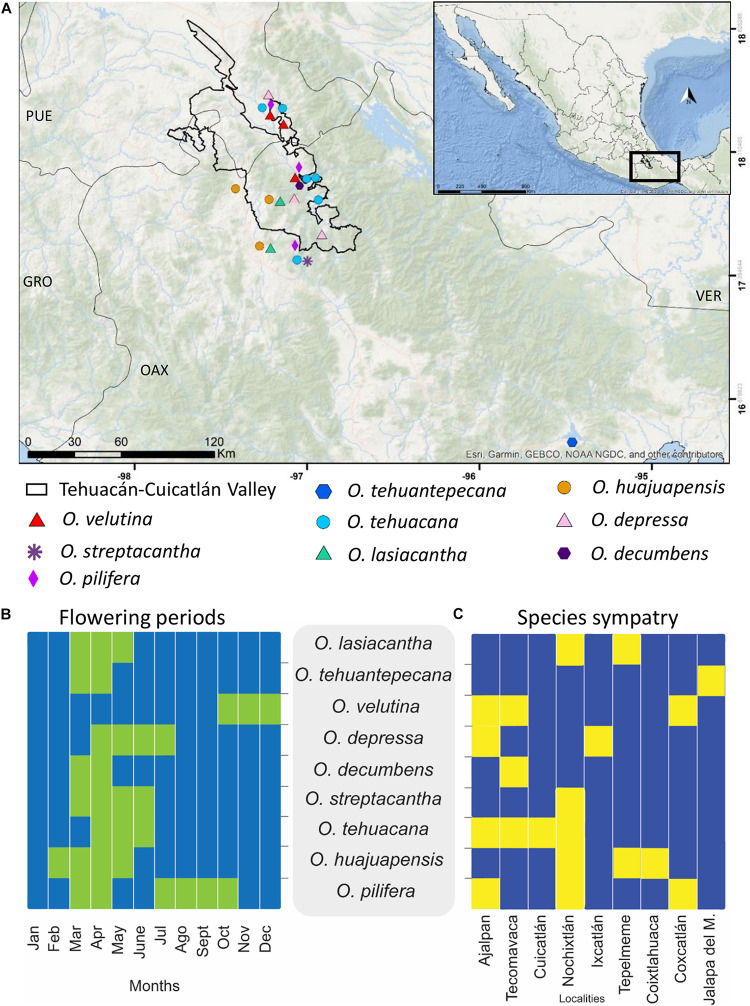
The *Opuntia* species from Tehuacán-Cuicatlán Valley and other species collected in Mexico evaluated on this work. **(A)** Sampled localities in field trips. **(B)** Heatmap showing in green overlap of flowering months between sampled species, data from Arias et al. (2012). **(C)** Heatmap showing in yellow species sympatry per sampled locality.

We sampled wild *Opuntia* species, which occur sympatrically with *O. tehuacana* and *O. pilifera* in the Tehuacán-Cuicatlán Valley (Arias et al., 2012), as well as additional species from the nearby Isthmus of Tehuantepec, Oaxaca, and El Arenal, Hidalgo, Mexico. As outgroup, *Grusonia invicta* was included based on phylogenetic relationships in Opuntioideae according to previous studies (Griffith and Porter, 2009; Guerrero et al., 2019). Plant material was collected through a series of field trips to Tehuacán-Cuicatlán Valley performed from October 2017 to June 2018. Additional plant material was obtained from the Cactaceae collection at Jardín Botánico, Instituto de Biología, Universidad Nacional Autónoma de México (UNAM). To ensure the identification and voucher ID of samples at Botanical Garden, sampling of this material was supervised by the Cactaceae specialist Ph.D. SA. In total nine *Opuntia* species were sampled and one to three individuals per species were included, in order to represent the variation of the species throughout its distribution. For the putative hybrids, we included three *O. pilifera* individuals and six *O. tehuacana* individuals. The sampled localities are shown in Figure 2A. Voucher specimens were deposited at Jardín Botánico, Instituto de Biología, UNAM and MEXU herbarium. Further collection and locality details are shown in Supplementary Table 1.

### Nuclear Marker Design

**FIGURE 3:**
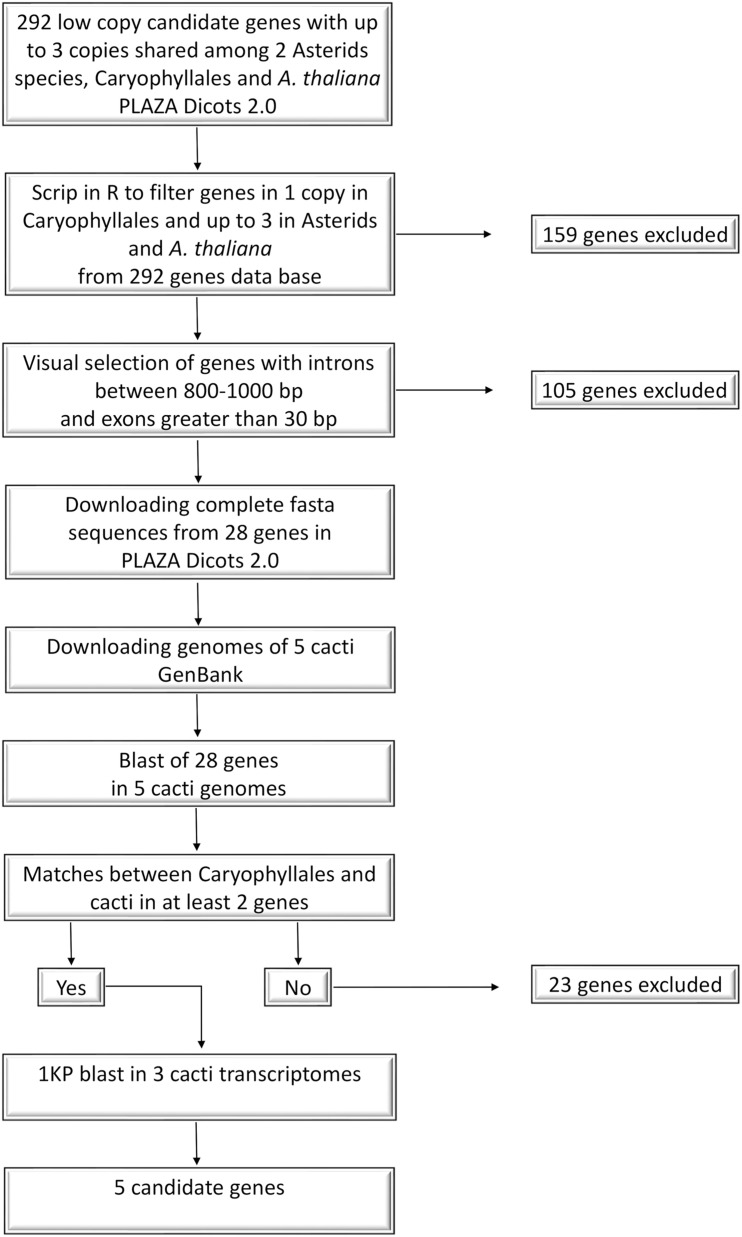
Transcriptome and genome mining workflow.

Our objective was to identify single copy nuclear markers potentially useful in Cactaceae species, applying a modification of the mining strategy proposed by Granados Mendoza et al. (2015). Our marker design workflow is summarized in Figure 3. Using the PLAZA v.4.0 dicots database^1^, we first compiled a file with a list of nuclear genes present in low copy in three representatives of the order Caryophyllales (*Amaranthus hypochondriacus*, *Chenopodium quinoa*, and *Beta vulgaris*), as well as two representatives from the Asterids clade (*Daucus carota* and *Actinidia chinensis*) and the model plant *Arabidopsis thaliana* (Order Brassicales, rosid clade). We wanted to obtain a greater number of candidate genes despite the fact that in Asterids and *A. thaliana* could have genes with more copies so, we selected gene families that were present in one copy in Caryophyllales and up to three copies on these distant lineages. Resulting in 292 low copy candidate genes, then, we filtered out those genes that were absent in Asterids and *A. thaliana* with an in-house R script (available at https://github.com/cristoichkov/Plaza_filter), retaining 133 candidate genes. To increase sequence variability, markers were designed to span more non-coding than coding regions. Because of this, genes without introns were excluded by visualizing each retained gene family model in the PLAZA v.4.0 dicots database. A total of 28 genes with introns were retained, and the complete and coding sequences of the Caryophyllales representatives were downloaded in fasta format and subsequently aligned in PhyDE v.0.9951 (Müller et al., 2005). Genes with introns between 800 and 1,000 base pairs (bp) and exons greater than 30 bp were further selected. To enrich Cactaceae species representation in our alignments and to improve primer design, we extracted orthologous sequences from the complete genomes of *Carnegiea gigantea*, *Lophocereus schottii*, *Pachycereus pringlei*, *Pereskia humboldtii*, and *Stenocereus thurberi* (NCBI Sequence Read Archive: SRR5036292 to SRR5036296 and SRR5137211 to SRR5137214, respectively; Copetti et al., 2017) with BLAST Command Line Tools v.2.7.1 (Camacho et al., 2009), using as reference the sequences from 28 selected genes. We selected and manually aligned in PhyDE the Caryophyllales genes that matched with at least two Cactaceae genomes. Five candidate genes were recovered, *AT3G05090*, *AT4G24040*, *AT3G48380*, *AT1G18270*, and *AT1G36980* (names based on the *A. thaliana* annotation). Additional Cactaceae species coding sequences from these five genes were mined from oneKP^2^ using BLAST in Geneious v.11.1.5 with the transcriptome databases of *Lophophora williamsii*, *Opuntia polyacantha*, and *Pereskia aculeata*, with matches for all genes. Scripts and more detailed information are available at GitHub repository^3^.

Finally, primers were designed only on the first four candidate genes (Table 1) due to the risk of sequences not overlapping in gene *AT1G36980* because its intron size of more than 1,200 bp for Cactaceae species. We designed up to three pairs of primers per candidate gene to span introns from 800 to 1,200 bp using the online eurofins primer design tool^4^.

**TABLE 1:** Nuclear primers designed on intron-flanking exons from selected genes.

Gene	Primer ID	Forward sequence 5′–3’	Reverse sequence 5′–3’	Tested
*AT3G05090*	a	TGACGGGACATTGAAGAGG	GTGGCTCCAGTTTCTAAGTC	Yes
	b	TAGCTACTTGCTCCGCTAC	GTCTAGGTGGGGAGGTTTT	No
	c	GGGCTTCTCTTGAAGGATCTAC	GTGGTGATTCAAGATTATGGCG	No
*AT4G24040*	a	AGAAATCTCTTCCTGCTCTAC	GACAACATTGGCTACTACATC	Yes
	b	AATGGTGCCAGAGCTTATTAC	AGAAATCTCTTCCTGCTCTAC	No
	c	GACAACATTGGCTACTACATC	GTTTGTGCTGCTGGGATTGC	No
*AT3G48380*	a	GTCTTACACCCRGTATCARTT	TATTGCATCTCTGGTTCAAGG	Yes
	b	CMGCAATGAAGAATGCAGTCT	GTCTTACACCCRGTATCARTT	No
*AT1G18270*	a	CAGTCTGATGCARTAGCRAGA	CCACCTTGTTGCTTCAGTTGA	Yes
	b	ACGAGGTGAGCACATGAAGCA	TCTGCCACTTCTTGGAGTTGC	No
	c	AACACTYTCTAGATTCGTGGA	ACCAATGAAGCTCAAGCAGAA	No

### Nuclear Primer Validation

Total genomic DNA was extracted from silica gel dried tissue following the CTAB protocol (Doyle and Doyle, 1987) with some modifications as reported by Bustamante et al. (2016) to avoid excess mucilage in the samples. DNA quality was tested with NanoDrop 2000. The DNA quality obtained with these modifications was good enough for Sanger sequencing. For each selected nuclear gene, the most promising pair of primers were selected according to previous cacti alignments (Table 1; primer ID “a”). To test the feasibility of primer amplification among Cactaceae species, we selected species from distant phylogenetic clades, namely: *Opuntia pilifera*, *O. tehuacana*, *Grusonia invicta*, *Pilosocereus chrysacanthus*, *P. collinsii*, *Melocactus curvispinus*, *Mammillaria albilanata* subsp. *oaxacana*, *M. haageana* subsp. *Meissneri*, and *M. crucigera*. We performed 15 μL PCRs with the commercial mix “Platinum Taq” (Invitrogen), and the reactions included 1.5 μL (1×) of 10× PCR buffer, 0.3 μL of dNTP mix, 0.3 μL of each primer (10 pmol/μL), 0.3 μL of BSA (0.4%), 0.6 μL of MgCl_2_ (1.5 μM), and 0.075 μL (0.375 units) of Taq DNA polymerase. Amplification tests were made using the touch-up PCR program (Table 2) as well as gradient PCR with two MgCl_2_ concentrations 1.5 and 2.5 μM (Table 3). To confirm the presence of PCR amplicons, these were run on 1% agarose gels. PCR cleaning and sequencing was performed at Laboratorio de Biología Molecular de la Biodiversidad y de la Salud, Instituto de Biología, UNAM, for sample sequencing the reactions included 0.4 μl of BigDye Terminator v.3.1 (Applied Biosystems), 2 μl of Buffer 5×, 4 μl of water, 1 μl of primer with a concentration of 10 μM and 3 μl of PCR product. Conditions of reaction sequencing were 30 cycles of 96°C for 10 s, 50°C for 5 s and 60°C for 4 min. After cycling, samples were purified with Centri-Sep (Thermo Fisher Scientific) plates following the manufacturer protocol. To each purified sample was added 25 μl of EDTA 0.5 mM and were run in a sequencer Applied Biosystems (Thermo Fisher Scientific) with polymer 7 (Thermo Fisher Scientific).

**TABLE 2:** Touch-up PCR program for amplification tests and primer ID “a” in *AT3G48380*.

	Temperature	Time	
Initial denaturation	94°C	2 min	
Denaturation	94°C	35 s	10 cycles
Annealing	46–51°C increasing 0.5°C per cycle	30 s	
Extension	72°C	1 min	
Denaturation	94°C	35 s	30 cycles
Annealing	55°C	30 s	
Extension	72°C	1 min	
Final extension	72°C	5 min	

**TABLE 3:** PCR program for gradient test and primer ID “a” in *AT1G18270*, using a concentration of 2.5 μM MgCl_2_.

	Temperature	Time	
Initial denaturation	94°C	2 min	
Denaturation	94°C	30 s	32 cycles
Annealing gradient	46–57°C	30 s	
Annealing *AT1G36980*	51.5°C		
Extension	72°C	1.5 min	
Final extension	72°C	5 min	

Primer pairs amplifying a single band in representatives of *Opuntia* and *Grusonia* were further amplified for this work. Sequences were assembled in Sequencher v.5.4.6. Alignments were performed with the program Muscle v.3.8.31 (Edgar, 2004) and subsequently manually adjusted in PhyDE (Müller et al., 2005).

### Phylogenetic and Network Analysis

We further amplified the primers with ID “a” for *AT3G48380* and *AT1G18270* genes (Table 1) in 26 individuals from the nine *Opuntia* species (Figure 1) and the outgroup. PCR amplification followed conditions on Tables 2, 3, respectively.

Phylogenetic networks analyses aimed to identify found hybridization among sampled *Opuntia* species in two levels : all the 27 sampled taxa, and at the individual level in *O. pilifera* and *O. tehuacana*. Therefore, we divided our data in two sets, one that included all 27 taxa and a second group indicated in Supplementary Table 1 as “hybrid-test,” which included only one random individual per species and one putative hybrid. In total we analyzed nine “hybrid-test” matrices with 10 taxa each. To perform the phylogenetic network analysis, we used as a base maximum likelihood (ML) gene trees from RA×ML v.8.2 (Stamatakis, 2014) with the GTR + Γ model, bootstrapping with the autoMRE option and a search for the best-scoring ML tree after the bootstrap searches to obtain the best tree.

The phylogenetic networks analysis was performed in PhyloNet v.3.6.1 (Wen et al., 2018) using the best ML trees for each data block. First, we tested the non-hybridization hypothesis in the complete data block (27 taxa), using the option Infer_ST_MDC to obtain the species tree under the “Minimize Deep Coalescence” (MDC) criterion; next we used all the available reticulation options for the InferNetwork_MP command to test the possible one, two, and three reticulation scenarios. Afterward, we performed nine individual tests for hybridization using each one of the nine “hybrid-test” matrices. For these tests, we first obtained the species trees (Infer_ST_MDC), and thereafter, we used the InferNetwork_MP with the option -h {putative hybrid} to test each individual hybrid hypothesis. For both analyses, each reticulation inference was replicated 10 times. In each one of the 10 replicates, the network with the lowest number of extra lineages was selected and displayed graphically with Dendroscope v.3.0 (Huson and Scornavacca, 2012). Furthermore, we analyze our complete matrix (without outgroup) in SplitsTree v.4.16.1, with a NeighborNet (Supplementary Figure 1) to identify potential reticulation between lineages with a non-parametric method.

### Testing Hybridization With Phylogenetic Invariants

We performed a phylogenetic invariants analysis under the coalescent model on HyDe v.0.4.1a (Hybridization Detection: Blischak et al., 2018) with the aim to test the following two hypotheses, (1) *O. tehuacana* as a hybrid between *O. pilifera* and *O. huajuapensis*, and (2) *O. pilifera* as a hybrid between species of the *Nopalea* and *Basilares* clades, as well as to confirm the resulting networks from PhyloNet. We used the data set of all 27 taxa first, with the command run_hyde.py to test 11 possible triplet combinations with the hybridization scenarios of *O. tehuacana*, *O. pilifera* and the resultant hybrid scenarios from PhyloNet, then we ran individual hybridization detection analyses (individual_hyde.py) with the file of resultant positive values, to detect hybridization at individual level.

To strengthen our analysis, we performed another hybridization analysis using Dsuite. We used as base the all 27 taxa alignment in Fasta format and we transformed it into VFC in Python v.3.8 with the library cflib-pomo v.1.2.2.1. Then we filtered this file with VCFtools v.0.1.17 to have only biallelic SNPs. After that we obtained 1,000 SNPs which we analyzed with the Dtrios option, using the same 11 possible hybridization scenarios tested in HyDe.

## Results

### Amplified Nuclear Genes and Data Matrices

From the four pairs of primers tested, only two for intron regions in genes *AT3G48380* and *AT1G18270*, indicated with ID “a” (Table 1) presented a single visible PCR product on an agarose gel in *Opuntia, Grusonia, Melocactus*, and *Pilosocereus*.

The aligned complete data block consisted of 27 terminals and 1,976 aligned characters, 1,102 of which correspond to the *AT3G48380* intron and 874 characters corresponding to the intron *AT1G18270*. The reduced data block “hybrid-test” consisted of nine data matrices with 10 terminals in each one and 1,976 aligned characters.

### Phylogenetic Networks From Coalescent-Based Methods

**FIGURE 4:**
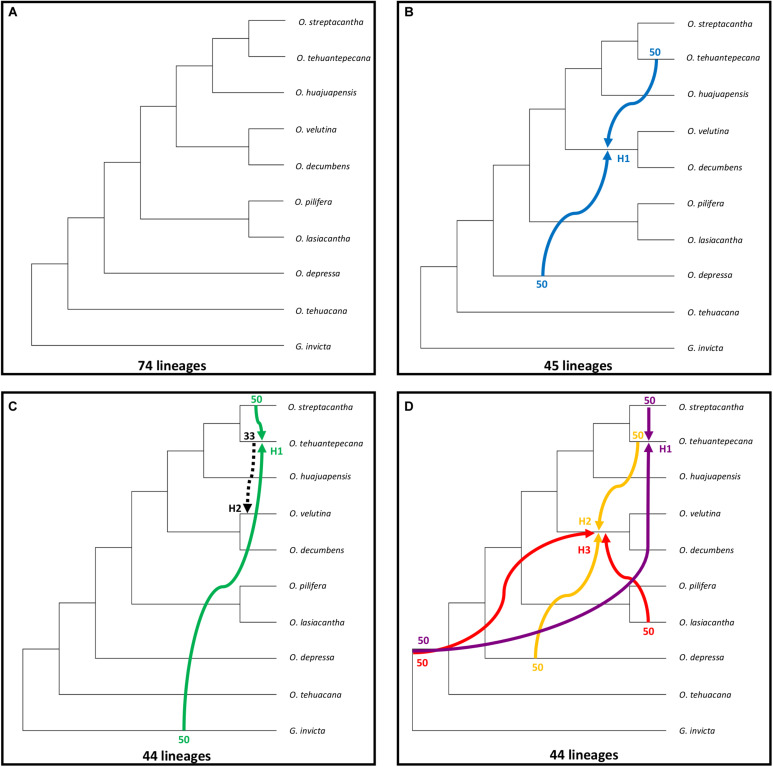
MDC analysis for all sampled *Opuntia* species. **(A)** Species tree without reticulations. **(B–D)** Best reticulation inference from one to three possible reticulations, number of reticulations indicated with H1, H2, H3. Inheritance probabilities are indicated before each arrow. Hybridization events are indicated with solid colored arrows, and the dashed arrow indicates an introgression event.

Under parsimony the MDC criterion seeks the reconciliation of a set of gene trees in the branches of a species tree with the minimal number of extra lineages, providing an optimal evolutive history for the species tree (Yu et al., 2013). We first inferred the species tree using the MDC criterion for the 27 sampled species, and the species tree had 74 lineages (Figure 4A), *O. tehuacana* and *O. depressa* were recovered as successive sister species of a clade containing the remaining sampled *Opuntia* species. Within the latter clade, two main groups were recovered. The first of them was composed of *O. pilifera*, sister to *O. lasiacantha*, and the second was integrated by a clade of *O. velutina*, sister of *O. decumbens*, and a clade where *O. tehuantepecana* is sister to the clade of *O. streptacantha* plus *O. huajuapensis*.

When inferring reticulation events for all sampled species we explored all the possible scenarios with one, two, and three reticulation events, for a total of six possible reticulation events. For the one reticulation event (Figure 4B; H1), PhyloNet detected hybridization between *O. tehuantepecana* and *O. depressa* into the clade *O. velutina-O. decumbens* and a reduction of 74 to 45 lineages. In the two reticulation event (Figure 4C), we detected hybridization between *O. streptacantha* and *G. invicta* into *O. tehuantepecana* (H1) and introgression from *O. tehuantepecana* into *O. velutina* (H2) with a reduction from 74 to 44 lineages. The three reticulations scenario (Figure 4D) resulted only in three hybridization events, of which the first (H1) occurred between *O. streptacantha* and a non-sampled or possible extinct taxon into *O. tehuantepecana*, the second (H2) involved *O. tehuantepecana* and *O. depressa* as a putative parental species for the *O. velutina-O. decumbens* clade and the third (H3) indicated hybridization between *O. lasiacantha* and a non-sampled or possibly extinct taxon into the *O. velutina-O. decumbens* clade. The lineage number remained the same as the two-reticulation event.

**FIGURE 5:**
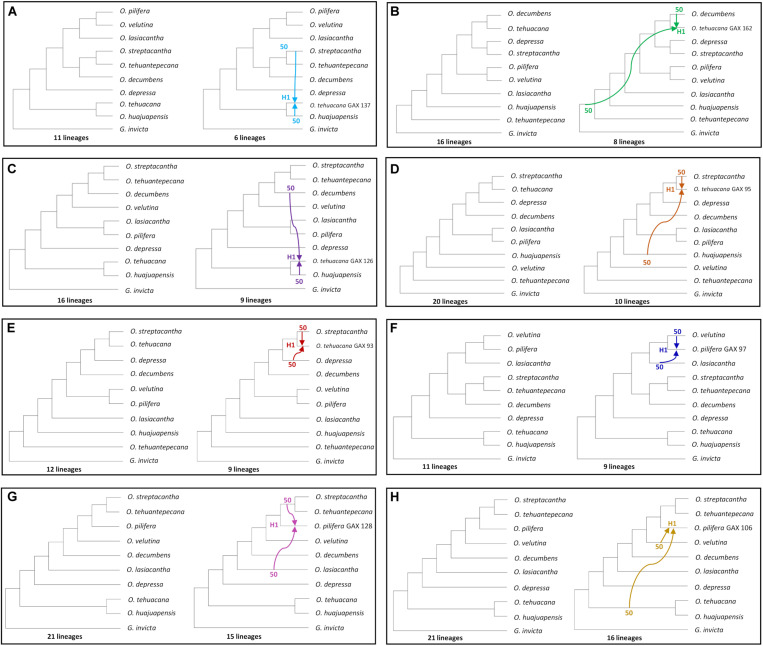
All individual hybridization tests made with MDC; on the left side is the species tree, and on the right side is the inferred reticulation with specific putative hybrids. Inheritance probabilities are indicated before each arrow. **(A–E)** *O. tehuacana* best MDC network. **(F–H)** *O. pilifera* best MDC network.

For the two hybrids hypothesis test, we sequentially indicate *O. tehuacana* and *O. pilifera* individuals as putative hybrids (Figures 5A–H). The first reticulation event (Figure 5A) showed 11 lineages for the MDC tree and hybridization (H1) between *O. streptacantha* and *O. huajuapensis* into *O*. *tehuacana* from Santa Maria Tecomavaca, Oaxaca, and a reduction to six lineages. The other *O. tehuacana* individual from this locality did not present a logical reticulation event. The following reticulation event (Figure 5B) depicts the MDC tree (16 lineages) and a hybridization event (H1, eight lineages) between *O. decumbens* and a non-sampled or extinct taxon into *O. tehuacana* from San Juan Bautista Cuicatlán, Oaxaca. The next MDC tree (Figure 5C) has 16 lineages, and it shows hybridization between *O. decumbens* and *O. huajuapensis* (H1) into *O. tehuacana* from Asunción Nochixtlán, Oaxaca. The following analysis was performed in *O. tehuacana* individuals from Ajalpan, Puebla, and the MDC tree (Figure 5D) has 20 lineages and depicts hybridization between *O. streptacantha* and *O. huajuapensis* and a reduction to 10 lineages. Afterward, the MDC tree (Figure 5E) depicts 12 lineages and a hybridization event between *O. streptacantha* and *O. lasiacantha* into *O. tehuacana* from Ajalpan, Puebla, with a reduction to nine lineages.

For *O. pilifera* hybridization tests (Figures 5F–H), the first analysis depicts the MDC tree with 11 lineages and a hybridization event that occurred between *O. velutina* and *O. lasiacantha* into *O. pilifera* from Ajalpan, Puebla and a reduction to nine lineages. In the following test (Figure 5G), the MDC tree has 21 lineages and a hybridization event between *O. lasiacantha* and the clade *O. streptacantha*-*O. tehuantepecana* into *O. pilifera* from Asunción Nochixtlán, Oaxaca. The last inferred MDC tree (Figure 5H) depicts 21 lineages and hybridization between *O. velutina* and the clade *O. tehuacana*-*O. huajuapensis* into *O. pilifera* from Ajalpan, Puebla.

### Corroboration of Phylogenetic Networks With HyDe and Dsuite

We tested 11 triplets with the command run_hyde.py, and the only significative triplet combination was *O. tehuantepecana*-*O. depressa* as parental for *O. velutina*. Although for the rest of the triplet combinations we had no significative results, we used the triplets with positive values (Table 4) to analyze hybridization at the individual level. We consider the results from individual analysis with a *p*-value lower than 0.05 important and reliable even though they were not significative because we include multiple individuals per species (Blischak et al., 2018). The individual analysis revealed six hybrid individuals with *p*-values lower than 0.05 (Table 5).

**TABLE 4:** Results from hybridization detection in HyDe with positive values.

P1	Hybrid	P2	Z-scores	*p-*values	Gamma (γ)
*O. decumbens*	*O. pilifera*	*O. depressa*	0.446	0.327	0.572
*O. lasiacantha*	*O. pilifera*	*O. velutina*	0.642	0.260	0.351
*O. decumbens*	*O. tehuacana*	*O. huajuapensis*	1.122	0.130	0.545
*O. tehuantepecana*	*O. velutina*	*O. depressa*	2.066	0.019	0.484
*O. tehuantepecana*	*O. decumbens*	*O. depressa*	1.093	0.137	0.310
*O. tehuantepecana*	*O. velutina*	*O. decumbens*	1.574	0.057	0.374

**TABLE 5:** Detected hybridization on individuals and their putative parental species in HyDe analysis (*p*-values lower than 0.05).

P1	Hybrid	P2	Z-score	*p*-value	Gamma (γ)
*O. decumbens*	*O. tehuacana* (GAX 162)	*O. huajuapensis*	1.720	0.042	0.686
*O. tehuantepecana*	*O. velutina* (GAX 108)	*O. depressa*	2.416	0.007	0.507
*O. tehuantepecana*	*O. velutina* (GAX 139)	*O. depressa*	2.008	0.022	0.460
*O. tehuantepecana*	*O. velutina* (GAX 96)	*O. depressa*	1.755	0.039	0.482
*O. tehuantepecana*	*O. decumbens* (GAX 134)	*O. depressa*	1.969	0.024	0.235
*O. tehuantepecana*	*O. velutina* (GAX 108)	*O. decumbens*	1.972	0.024	0.417

The same triplets tested on HyDe were used with the Dtrios command on Dsuite, and there were ten combinations with significative *p*-values (Supplementary Table 2). The combinations with shared alleles and supported by other analysis were *O. decumbens*-*O. pilifera*, *O. decumbens*-*O. tehuacana*, and *O. tehuantepecana*-*O. velutina*.

## Discussion

We tested two hybridization cases, as well as previously unknown hybridization scenarios involving sympatric Opuntia species from Tehuacán-Cuicatlán Valley and southern Mexico. Our findings support previous work, in which highlight Opuntia as a genus with multiple hybridization events (Pinkava, 2002; Majure et al., 2012a) but, in this work using new approaches.

We used a multi-individual approach in our analysis to compensate for the low number of nuclear sites (1,976) and obtain reliable results. Phylogenetic networks inferred for all the sampled species showed a lineage decrease compared to the species tree (Figure 4); therefore, the scenarios with inferred reticulations can be assumed to be more accurate (Wen et al., 2018). Some of the recovered reticulation scenarios include the outgroup, non-sampled or extinct taxa, we consider these scenarios unlikely, and also probably due to the retention of an ancestral polymorphism, therefore, we only mention them as part of PhyloNet results (Copetti et al., 2017; Wen et al., 2018). Consequently, we emphasize the significance of having a broader sampling for future analysis, to avoid this kind of implausible scenarios. In *Opuntia*, the presence of natural hybrids due to weak reproductive barriers is common (Pinkava, 2002). The flowering periods for most of the studied opuntias occur during the spring (Figure 2B); hence, pollen exchange could occur between geographically close species. The pollination is mainly carried out by bees known for being generalists favoring cross-pollination between different species (Reyes-Agüero et al., 2006), and there is even evidence of pollination by hummingbirds in the Tehuacán-Cuicatlán Valley, carrying pollen across wider distances than bees (Ortiz-Pulido et al., 2011; Serrano-Serrano et al., 2017).

### Implications of Sympatry in Studied Opuntias

It is important to emphasize that most of the sampled species for this study are in sympatry (Figure 2C), but we mean not overlapping in potential geographic distributions but rather three to four species inhabiting the same small area. For example, in Ajalpan, Puebla, the species *O. tehuacana*, *O. pilifera*, *O. velutina*, and *O. depressa* coexist in an area of approximately 20 m. This distribution dynamics allows the development of hybrids because of the pollen exchange (Reyes-Agüero et al., 2006) and weak mating barriers (Pinkava, 2002; Majure et al., 2012b). Although we did not formally study ecological dynamics of sampled opuntias we can made some inferences about exchange of genetic material based on our field observations, results, and data from literature about flowering periods (Arias et al., 2012). Hybrid zones in nature are the spatially and temporally place where two distinguishable populations overlap and cross to form viable and sometimes fertile offspring (Arnold, 1997), therefore we can say that most of the sampled localities could be considered as potential hybrid zones. In the cases of the outgroup and additional species like *O. tehuantepecana* and *O. streptacantha*, for which we include sampled individuals geographically distant from Tehuacán-Cuicatlán Valley, we analyzed them to know their relationships with the rest of the species and to have a hybridization context potentially older and wider.

### Hybridization and Gene Flow on *O. tehuacana*

The hypothesis of hybrid status in *O. tehuacana* described in previous studies by Arias et al. (2012), was not supported in any individual network. The only scenario that includes one of the proposed parental species occurs in one individual from Ajalpan, Puebla (X. Granados 95; Figure 5D). The putative parental species for this individual are *O. streptacantha* and *O. huajuapensis*, which are sister species (Figure 5D); however, we did not confirm *O. streptacantha* as a parental with the HyDe or Dsuite analysis, resulting in an implausible scenario. Regarding the reticulation event from *O. huajuapensis* into *O. tehuacana* from Ajalpan, introgression could exist because their floral periods overlap (April–May), their flowers are yellow, sometimes orange in the case of *O. tehuacana*, and there is evidence that the same species of hummingbird visits both opuntias species (Ortiz-Pulido et al., 2011; Arias et al., 2012), making pollen exchange between these species likely. Additional morphological characteristics shared between these species (Figures 1A,C) that could support the genetic exchange are the orbicular to suborbicular cladodes and the acid pulp in their fruits (Arias et al., 2012). Despite these shared traits, we did not observe intermediate characters in sampled *O. tehuacana* individuals, as would be expected in a hybrid (Arnold, 1997).

Another proposed parental species for *O. tehuacana* is *O. pilifera* (Arias et al., 2012), but for this scenario, no reticulation event was found either in PhyloNet, HyDe or Dsuite tests. Both species (Figures 1A,E) share the presence of hairs in the areolas (sporadic in *O. tehuacana*) and glabrous epidermis. However, *O. tehuacana* is a shrub, the flower is typically orange–yellow and the fruit can remain on the plant for more than a year until it becomes green–yellow, with acidic, light pink pulp. Meanwhile, *O. pilifera* is a tree, the flower is red–pink and the fruit remains on the plant for only one season, turning red to light pink, with sweet and red pulp. Since there are no intermediate traits between these species or another result supporting the parental scenario of *O. pilifera*, this hypothesis is discarded (Arnold, 1997; Reyes-Agüero et al., 2006).

We tested the hypothesis of *O. huajuapensis* and *O. decumbens* as parental species of *O. tehuacana* in order to support the hybridization scenario obtained from PhyloNet (Figure 5C). This scenario involves the *O. tehuacana* individual from Nochixtlán. Surprisingly, from the six *O. tehuacana* individuals tested on HyDe, reliable hybridization was detected only in the specimen from Cuicatlán (X. Granados 162), and the reticulation analysis for this individual (Figure 5B) involves *O. decumbens* and a non-sampled or extinct taxon. Therefore, we think that the reticulation event between these species was influenced by the fact that these individuals are phylogenetically close, but in fact this relationship is only present on this MDC tree. It is important to emphasize that although HyDe did not detect hybridization in *O. tehuacana* from Nochixtlán, the same gene flow pattern could be shared between individuals from Cuicatlán and Nochixtlán because *O. tehuacana* and *O. huajuapensis* are sympatric in both places (Arias et al., 2012). The pattern of reticulation from *O. decumbens* into *O. tehuacana* appears twice in the inferred individual networks, was recovered in one individual HyDe analysis, supported by Dsuite analysis in two individuals (Supplementary Table 2) and was also found in the NeighborNet (Supplementary Figure 1). The floral periods of *O. decumbens* and *O. tehuacana* occur during the same period (Figure 2B) and they are sympatric thus, cross pollination can occur between analyzed individuals from both species. Our results confirm that not all *O. tehuacana* individuals have the same reticulation pattern, and we can infer that only introgression among certain individuals is occurring and not hybrid speciation (Blischak et al., 2018).

### Hybridization and Gene Flow on *O. pilifera*

*Opuntia pilifera* is in the *Basilares* clade (Majure et al., 2012a), species of this clade are known for being polyploids and form hybrids. In the analysis performed on PhyloNet with the data set of all individuals, we did not detect hybridization into *O. pilifera* in any inferred network scenario, which could also be related to the low number of loci used and insufficient sampling of closely related species in our study. On the other hand, the test performed on *O. pilifera* individuals revealed hybridization scenarios involving mainly *O. lasiacantha* and *O. velutina*. The pattern of *O. velutina*–*O. pilifera*, and *O. lasiacantha*–*O. pilifera* as sister species is repeated several times on species trees from Figures 4, 5. The changing position of these species could also support the hybridization scenarios obtained in the PhyloNet individual analysis (Wen et al., 2018). Furthermore, in the HyDe analysis to test *O. pilifera* as a hybrid, we obtained positive values (Table 4) for the putative *O. pilifera* parental lineages *O. decumbens*–*O. depressa* and *O. lasiacantha*–*O. velutina*, but when we performed the individual analysis, none of the individually tested triplets had a reliable *p*-value; thus, the HyDe analysis cannot confirm the results obtained in PhyloNet. Surprisingly, the analysis performed on Dsuite supports the scenario of *O. decumbens* as parental donor for *O. pilifera*. This sustains the hybridization hypothesis proposed by Majure et al. (2012a), because *O. decumbens* is in clade *Nopalea*. Furthermore, the bloom of these species overlaps and they are sympatric on Tehuacán-Cuicatlán Valley (Figure 2B; Arias et al., 2012).

### Hybridization in Opuntias From Southern Mexico

In the phylogenetic network analysis with all sampled species, the one reticulation event (Figure 4B) depicts hybridization into the *O. velutina*–*O. decumbens* clade with putative parental species *O. tehuantepecana* and *O. depressa*. This scenario is also supported by HyDe analysis with significative hybridization between *O. depressa* and *O. tehuantepecana* intro *O. velutina* (Table 4) and at individual level all sampled *O. velutina* and one individual from *O. decumbens* had reliable hybridization results (Table 5). The analysis with Dsuite also supports the relationship of *O. tehuantepecana* as parental of *O. velutina* (Supplementary Table 2). In the hybridization scenario that includes *O. tehuantepecana* and *O. depressa* as putative parental lineages of *O. velutina*, we infer that the event probably occurred in the past because current distributions of parental species are adjacent but not sympatric (Arias et al., 2012; Barthlott et al., 2015). The *O. depressa* distribution was probably wide enough to contact *O. tehuantepecana*, giving rise to a hybrid zone in which a lineage with similar fitness to its parental species originated after backcrosses and the action of natural selection (Arnold, 1997), which is currently known as *O. velutina*. This assumption is also supported by morphological similarities shared by *O. velutina* and *O. tehuantepecana* (Figures 1B,I): both are shrubs, sometimes tree-like with a wide trunk; their glochids are long and yellow, from 5 to 13 mm in *O. velutina* and from 2 to 4 mm in *O. tehuantepecana*; and the traits shared between *O. velutina* and *O. depressa* are the cladodes obovate and pubescent and glochids long and yellow (Anderson, 2001; Arias et al., 2012). The relationship among these species have not been reported elsewhere.

More introgression scenarios between analyzed *Opuntia* species were recovered with the Dsuite analysis, but most of them involve *O. tehuantepecana* and one specie from Tehuacán-Cuicatlán Valley, since these species are not sympatric, the number of SNPs analyzed was too low to assign the true parental donor (Malinsky et al., 2020) and the scenarios were not recovered in other analysis, we consider them unlikely.

### Taxonomic Implications of Hybridization in *Opuntia*

The hybridization process in *Opuntia* has evolutive implications on the number of species, the success and survival of his taxa in habitats with extreme weather conditions, but also has significance on the phylogenetic relationships within this group (Pinkava, 2002; Majure et al., 2012a). Not having a bifurcate history complicates the understanding of a group under classical phylogenies where the inheritance pattern is linear (Arnold, 1997). The horizontal exchange of genetic material in opuntias hinders their linear phylogenetic histories and makes the limits among species blurred. *Opuntia* is one of the most complex groups of plants, due to its variable traits, polyploidy, hybridization, and human handling (Pinkava, 2002; Reyes-Agüero et al., 2006). Furthermore, the widespread distribution of species like *O. decumbens*, *O. streptacantha*, and *O. velutina* complicates the collection of multiple individuals throughout its distribution and their inclusion in phylogenetic and network analyzes. New perspectives of reticulate evolution in plants should lead to an integrative vision with sampling multiple individuals, morphometric and biogeographic analysis, and other studies to improve taxonomy of problematic groups.

As future perspectives, we highlight the relevance of this work as a first approach to the hybridization processes in southern Mexico *Opuntia* species, a group that has been little studied despite the large number of species in this region (Arias et al., 2012). Phylogenetic approaches have included some *Opuntia* representatives from the Tehuacán-Cuicatlán Valley (Griffith and Porter, 2009; Majure et al., 2012a), but for most of the representatives of this complex group their evolutive stories remain unknown. This is mainly due to polyploidy and unknown chromosome numbers of species like *O. depressa*, *O. huajuapensis*, *O. tehuacana*, *O. tehuantepecana*, and *O. velutina*. Also, the allopolyploid condition in most *Opuntia* species hinders their study due to the presence of homoeologous genes, which are difficult to isolate and to distinguish from paralogous in the subgenomes (Glover et al., 2016).

New hybridization scenarios were found, as expected, in sympatric opuntias such as *O. velutina* as a putative hybrid. Its variable morphological traits and broad distribution (Bravo-Hollis, 1978) make this species an interesting study case for future analysis. Other hybridization scenarios were recovered in our analyses, but we do not have enough information to confirm them.

The case of *O. tehuacana* as a hybrid between *O. pilifera* and *O. huajuapensis* was discarded by our analysis, but due to hybridization detected in some individuals with *O. huajuapensis* and *O. decumbens* as the parental species, this scenario should be tested using more loci and including morphological and morphometric analysis because of the complex relationships among these species. The hybrid status of *O. pilifera* involves two pairs of putative parental species, *O. lasiacantha*–*O. velutina* and *O. decumben–O. depressa*. The relationship of *O. decumbens* as parental donor of *O. pilifera* was recovered as significative by the analysis of Dsuite, supporting the hypothesis of gene flow between *Nopalea* and *Basilares* clades. Other mentioned putative parental donors should be tested in further analysis.

Although PhyloNet results may not be significative due to the low number of genes used, we performed a multi-individual approach to compensate this disadvantage, and each individual was tested in a hybridization scenario allowing us to detect logical hybridization scenarios, hybrid individuals and introgression. Future studies in *Opuntia* should include more individuals per species and more loci, and, most importantly, carry out integrative analyses that allow elucidation of the reticulate evolution of this complex group of plants with high diversity in Mexico.

## Data Availability Statement

The datasets presented in this study can be found in online repositories. The names of the repository/repositories and accession number(s) can be found in the article/Supplementary Material.

## Author Contributions

XG-A: investigation, formal analysis, methodology, writing the first draft, text revision, and editing. CGM: methodology conceptualization, text revision, and editing. CC: methodology and complementary analysis. JM: methodology conceptualization, formal analysis, text revision, and editing. SA: researcher leading of this study and obtained the financial support. SA and XG-A: designing the research. All of the authors approved the submitted version of this manuscript.

## Conflict of Interest

The authors declare that the research was conducted in the absence of any commercial or financial relationships that could be construed as a potential conflict of interest. The reviewer LM declared a past co-authorship with one of the authors SA to the handling editor.

## Footnotes

1 https://bioinformatics.psb.ugent.be/plaza/versions/plaza_v4_dicots/

2 https://sites.google.com/a/ualberta.ca/onekp/

3 https://github.com/cristoichkov/Plaza_filter

4 https://www.eurofinsgenomics.eu/en/ecom/tools/pcr-primer-design/

## Supplementary Material

Supplementary Figure 1 | Neighbor net for 26 *Opuntia* individuals from 9 species.
