# Supplementary material for: Unraveling Reticulate Evolution in Opuntia (Cactaceae) From Southern Mexico
Source: Front Plant Sci. 2021 Jan 13;11:606809. doi: 10.3389/fpls.2020.606809 (PMC7838128; doi:10.3389/fpls.2020.606809)
Supplement: Supplementary file 1 [file Table_1.DOCX]

**Supplementary Table 1.** Sampled species and GenBank accession numbers for nuclear markers *AT3G48380* and in bold to *AT1G18270*.

| Specie | Collection data | State | Municipality | Group | GeneBank accession |
| --- | --- | --- | --- | --- | --- |
| *Grusonia invicta* | WLF s/n | UNAM Botanic Garden Cactaceae collection | | Outgroup | MT475832  **MT475859** |
| *Opuntia decumbens* | X. Granados 135 | Oaxaca | Santa María Tecomavaca |  | MT475834  **MT475861** |
| *Opuntia decumbens* | X. Granados 134 | Oaxaca | Santa María Tecomavaca | “hybrid-test“ | MT475835  **MT475862** |
| *Opuntia depressa* | X. Granados 130 | Oaxaca | San Pedro Ixcatlán |  | MT475837  **MT475864** |
| *Opuntia depressa* | X. Granados 133 | Oaxaca | San Pedro Ixcatlán |  | MT475833  **MT475860** |
| *Opuntia depressa* | X. Granados 98 | Puebla | Ajalpan | “hybrid-test“ | MT475836  **MT475863** |
| *Opuntia huajuapensis* | X. Granados 118 | Oaxaca | Tepelmeme Villa de Morelos | “hybrid-test“ | MT475839  **MT475866** |
| *Opuntia huajuapensis* | S. Arias 2135 | Oaxaca | San Juan Bautista Coixtlahuaca |  | MT475838  **MT475865** |
| *Opuntia huajuapensis* | X. Granados 123 | Oaxaca | Asunción Nochixtlán |  | MT475840  **MT475867** |
| *Opuntia lasiacantha* | X. Granados 121 | Oaxaca | Tepelmeme Villa de Morelos |  | MT475841 |
| *Opuntia lasiacantha* | X. Granados 122 | Oaxaca | Asunción Nochixtlán | “hybrid-test“ | MT475842  **MT475868** |
| *Opuntia pilifera* | X. Granados 97 | Puebla | Ajalpan | putative hybrid | MT475843  **MT475869** |
| *Opuntia pilifera* | X. Granados 128 | Oaxaca | Asunción Nochixtlán | putative hybrid | MT475845  **MT475871** |
| *Opuntia pilifera* | X. Granados 106 | Puebla | Coxcatlán | putative hybrid | MT475844  **MT475870** |
| *Opuntia streptacantha* | S/C 6 | UNAM Botanic Garden Cactaceae collection | |  | MT475846 |
| *Opuntia streptacantha* | X. Granados 140 | Hidalgo | El Arenal |  | MT475847  **MT475872** |
| *Opuntia streptacantha* | X. Granados 127 | Oaxaca | Asunción Nochixtlán |  | MT475848  **MT475873** |
| *Opuntia tehuacana* | X. Granados 137 | Oaxaca | Santa María Tecomavaca | putative hybrid | MT475852  **MT475877** |
| *Opuntia tehuacana* | X. Granados 150 | Oaxaca | Santa María Tecomavaca | putative hybrid | MT475853  **MT475878** |
| *Opuntia tehuacana* | X. Granados 126 | Oaxaca | Asunción Nochixtlán | putative hybrid | MT475851  **MT475876** |
| *Opuntia tehuacana* | X. Granados 162 | Oaxaca | San Juan Bautista Cuicatlán | putative hybrid | MT475854  **MT475879** |
| *Opuntia tehuacana* | X. Granados 93 | Puebla | Ajalpan | putative hybrid | MT475849  **MT475874** |
| *Opuntia tehuacana* | X. Granados 95 | Puebla | Ajalpan | putative hybrid | MT475850  **MT475875** |
| *Opuntia tehuantepecana* | X. Granados 170 | Oaxaca | Santa María Jalapa del Marqués | “hybrid-test“ | MT475855  **MT475880** |
| *Opuntia velutina* | X. Granados 96 | Puebla | Ajalpan |  | MT475856  MT475881 |
| *Opuntia velutina* | X. Granados 139 | Oaxaca | Santa María Tecomavaca |  | MT475858  MT475883 |
| *Opuntia velutina* | X. Granados 108 | Puebla | Coxcatlán | “hybrid-test“ | MT475857  MT475882 |

**Supplementary Table 2.** Results from Dsuite analysis, significative combinations in bold type.

| P1 | P2 | P3 | Dstatistic | Z.score | p.value | f_4_.ratio | BBAA | ABBA | BABA |
| --- | --- | --- | --- | --- | --- | --- | --- | --- | --- |
| *O. depressa* | *O. huajuapensis* | *O. decumbens* | 0.464567 | 1.14192 | 0.126744 | 0.345029 | 5.88889 | 5.16667 | 1.88889 |
| *O. depressa* | *O. lasiacantha* | *O. decumbens* | 0.016129 | 0.0497965 | 0.480142 | 0.0222222 | 9.08333 | 5.25 | 5.08333 |
| *O. decumbens* | *O. pilifera* | *O. depressa* | 0.5 | 1.32288 | 0.0929384 | 0.30303 | 5.61111 | 5 | 1.66667 |
| *O. depressa* | *O. streptacantha* | *O. decumbens* | 0.261146 | 0.616504 | 0.268781 | 0.414141 | 7.55556 | 5.5 | 3.22222 |
| ***O. depressa*** | ***O. tehuacana*** | ***O. decumbens*** | **0.644068** | **1.79582** | **0.0362619** | **0.36715** | **5.58333** | **5.38889** | **1.16667** |
| *O. depressa* | *O. decumbens* | *O. tehuantepecana* | 0.5 | 0 | NA | 0.25641 | 6.16667 | 5 | 1.66667 |
| *O. decumbens* | *O. velutina* | *O. depressa* | 0.677419 | 0 | NA | 0.291667 | 5.33333 | 4.33333 | 0.833333 |
| *O. lasiacantha* | *O. huajuapensis* | *O. decumbens* | 0.466667 | 1.56665 | 0.0585986 | 0.388889 | 7.77778 | 4.88889 | 1.77778 |
| *O. huajuapensis* | *O. pilifera* | *O. decumbens* | 0.176471 | 0.563099 | 0.286684 | 0.666667 | 5.66667 | 2.22222 | 1.55556 |
| *O. streptacantha* | *O. huajuapensis* | *O. decumbens* | 0.216 | 0.605932 | 0.27228 | 0.25 | 6.81481 | 2.81481 | 1.81481 |
| *O. huajuapensis* | *O. tehuacana* | *O. decumbens* | 0.320755 | 1.04104 | 0.148927 | 0.188889 | 5.94444 | 1.94444 | 1 |
| *O. decumbens* | *O. huajuapensis* | *O. tehuantepecana* | 0 | 0 | NA | 0 | 5.66667 | 1.22222 | 1.22222 |
| *O. huajuapensis* | *O. velutina* | *O. decumbens* | 0.407407 | 1.19277 | 0.11648 | 0.122222 | 3.22222 | 2.11111 | 0.888889 |
| ***O. lasiacantha*** | ***O. pilifera*** | ***O. decumbens*** | **0.53125** | **1.64577** | **0.0499051** | **0.472222** | **5.83333** | **5.44444** | **1.66667** |
| *O. lasiacantha* | *O. streptacantha* | *O. decumbens* | 0.351852 | 1.30618 | 0.0957451 | 0.234568 | 55.9444 | 4.05556 | 1.94444 |
| ***O. lasiacantha*** | ***O. tehuacana*** | ***O. decumbens*** | **0.634783** | **2.12913** | **0.0166219** | **0.675926** | **6.08333** | **5.22222** | **1.16667** |
| ***O. lasiacantha*** | ***O. decumbens*** | ***O. tehuantepecana*** | **0.657143** | **1.95224** | **0.0254548** | **0.283951** | **6.16667** | **4.83333** | **1** |
| *O. decumbens* | *O. velutina* | *O. lasiacantha* | 0.6 | 1.53093 | 0.0628932 | 0.0743802 | 5.33333 | 4 | 1 |
| *O. streptacantha* | *O. pilifera* | *O. decumbens* | 0.365854 | 1.03185 | 0.151072 | 0.555556 | 5.55556 | 3.11111 | 1.44444 |
| *O. pilifera* | *O. tehuacana* | *O. decumbens* | 0.102041 | 0.294566 | 0.384163 | 0.0793651 | 6.16667 | 1.5 | 1.22222 |
| *O. decumbens* | *O. pilifera* | *O. tehuantepecana* | 0.111111 | 0.226805 | 0.410288 | 0.0344828 | 6.44444 | 1.66667 | 1.33333 |
| *O. pilifera* | *O. velutina* | *O. decumbens* | 0.2 | 0.494975 | 0.310309 | 0.0584795 | 4.44444 | 1.66667 | 1.11111 |
| *O. streptacantha* | *O. tehuacana* | *O. decumbens* | 0.522388 | 1.49091 | 0.0679928 | 0.324074 | 5.83333 | 2.83333 | 0.888889 |
| *O. streptacantha* | *O. decumbens* | *O. tehuantepecana* | 0.352941 | 0 | NA | 0.121212 | 6 | 2.55556 | 1.22222 |
| *O. streptacantha* | *O. velutina* | *O. decumbens* | 0.526316 | 1.24827 | 0.105967 | 0.37037 | 3.88889 | 3.22222 | 1 |
| *O. decumbens* | *O. tehuacana* | *O. tehuantepecana* | 0.714286 | 0 | NA | 0.172414 | 5.72222 | 2 | 0.333333 |
| *O. tehuacana* | *O. velutina* | *O. decumbens* | 0.116279 | 0.283775 | 0.388291 | 0.119048 | 3.83333 | 1.33333 | 1.05556 |
| *O. decumbens* | *O. velutina* | *O. tehuantepecana* | 0.666667 | 0 | NA | 0.137931 | 6 | 1.66667 | 0.333333 |
| *O. huajuapensis* | *O. lasiacantha* | *O. depressa* | 6.83E-17 | 2.52E-16 | 0.5 | 3.81E-16 | 8.66667 | 6.5 | 6.5 |
| *O. pilifera* | *O. huajuapensis* | *O. depressa* | 0.130435 | 0.4136 | 0.339583 | 0.142857 | 6.27778 | 2.88889 | 2.22222 |
| *O. huajuapensis* | *O. streptacantha* | *O. depressa* | 0.0361446 | 0.134409 | 0.44654 | 0.047619 | 7.72222 | 4.77778 | 4.44444 |
| *O. huajuapensis* | *O. tehuacana* | *O. depressa* | 0.0931677 | 0.320478 | 0.374303 | 0.15625 | 6.25 | 2.44444 | 2.02778 |
| *O. depressa* | *O. huajuapensis* | *O. tehuantepecana* | 0.526316 | 0 | NA | 0.25641 | 10 | 4.83333 | 1.5 |
| *O. velutina* | *O. huajuapensis* | *O. depressa* | 0.0782609 | 0 | NA | 0.1 | 5.05556 | 3.44444 | 2.94444 |
| *O. pilifera* | *O. lasiacantha* | *O. depressa* | 0.0547945 | 0.19988 | 0.420787 | 0.25 | 6.75 | 6.41667 | 5.75 |
| *O. lasiacantha* | *O. streptacantha* | *O. depressa* | 0.0298507 | 0.121635 | 0.451594 | 0.0952381 | 55.5833 | 5.75 | 5.41667 |
| *O. lasiacantha* | *O. tehuacana* | *O. depressa* | 0.0344828 | 0.126655 | 0.449607 | 0.0595238 | 6.91667 | 6.25 | 5.83333 |
| *O. lasiacantha* | *O. depressa* | *O. tehuantepecana* | 0.047619 | 0.129099 | 0.448639 | 0.037037 | 14 | 5.5 | 5 |
| *O. lasiacantha* | *O. depressa* | *O. velutina* | 0.0266667 | 0.0930486 | 0.462932 | 0.0350877 | 6.91667 | 6.41667 | 6.08333 |
| *O. pilifera* | *O. streptacantha* | *O. depressa* | 0.126761 | 0.414921 | 0.3391 | 0.375 | 6.5 | 4.44444 | 3.44444 |
| *O. pilifera* | *O. tehuacana* | *O. depressa* | 0.364486 | 1.43325 | 0.0758928 | 0.191176 | 6.5 | 2.02778 | 0.944444 |
| *O. depressa* | *O. pilifera* | *O. tehuantepecana* | 0.578947 | 1.18177 | 0.118648 | 0.282051 | 9.16667 | 5 | 1.33333 |
| *O. pilifera* | *O. velutina* | *O. depressa* | 0.037037 | 0.124544 | 0.450442 | 0.0357143 | 6.11111 | 2.33333 | 2.16667 |
| *O. streptacantha* | *O. tehuacana* | *O. depressa* | 0.0110701 | 0.0440742 | 0.482423 | 0.0147059 | 6.61111 | 3.80556 | 3.72222 |
| *O. depressa* | *O. streptacantha* | *O. tehuantepecana* | 0.25 | 0.51031 | 0.304917 | 0.153846 | 11.8333 | 5 | 3 |
| *O. velutina* | *O. streptacantha* | *O. depressa* | 0.0877193 | 0.257576 | 0.398367 | 0.0833333 | 6 | 5.16667 | 4.33333 |
| *O. depressa* | *O. tehuacana* | *O. tehuantepecana* | 0.882353 | 0 | NA | 0.384615 | 9.25 | 5.33333 | 0.333333 |
| *O. velutina* | *O. tehuacana* | *O. depressa* | 0.204969 | 0.642249 | 0.260356 | 0.211538 | 5.27778 | 2.69444 | 1.77778 |
| *O. depressa* | *O. velutina* | *O. tehuantepecana* | 0.933333 | 0 | NA | 0.358974 | 8.16667 | 4.83333 | 0.166667 |
| *O. pilifera* | *O. huajuapensis* | *O. lasiacantha* | 0.277311 | 1.16049 | 0.122925 | 0.045082 | 5.44444 | 4.22222 | 2.38889 |
| *O. lasiacantha* | *O. streptacantha* | *O. huajuapensis* | 0.131579 | 0.477498 | 0.316504 | 0.20202 | 51.6667 | 4.77778 | 3.66667 |
| *O. tehuacana* | *O. huajuapensis* | *O. lasiacantha* | 0.183099 | 0.701178 | 0.241596 | 0.0336788 | 5.55556 | 3.5 | 2.41667 |
| ***O. lasiacantha*** | ***O. huajuapensis*** | ***O. tehuantepecana*** | **0.621622** | **1.74442** | **0.0405428** | **0.283951** | **12.3333** | **5** | **1.16667** |
| *O. lasiacantha* | *O. huajuapensis* | *O. velutina* | 0.297297 | 1.0726 | 0.141725 | 0.25731 | 5.88889 | 5.33333 | 2.88889 |
| *O. pilifera* | *O. huajuapensis* | *O. streptacantha* | 0.176471 | 0.563691 | 0.286482 | 0.152381 | 3.07407 | 2.96296 | 2.07407 |
| *O. pilifera* | *O. tehuacana* | *O. huajuapensis* | 0.302013 | 1.00887 | 0.156517 | 0.185185 | 2.46296 | 1.7963 | 0.962963 |
| *O. huajuapensis* | *O. pilifera* | *O. tehuantepecana* | 0.111111 | 0.248452 | 0.401892 | 0.0344828 | 9.88889 | 1.66667 | 1.33333 |
| *O. huajuapensis* | *O. pilifera* | *O. velutina* | 0.348837 | 1.18448 | 0.118112 | 0.204082 | 3.33333 | 3.22222 | 1.55556 |
| *O. tehuacana* | *O. huajuapensis* | *O. streptacantha* | 0.0124481 | 0.042292 | 0.483133 | 0.0114943 | 3.2037 | 2.25926 | 2.2037 |
| *O. streptacantha* | *O. huajuapensis* | *O. tehuantepecana* | 0.333333 | 0.745356 | 0.228028 | 0.121212 | 11.1111 | 2.66667 | 1.33333 |
| *O. streptacantha* | *O. huajuapensis* | *O. velutina* | 0.0769231 | 0.247964 | 0.402081 | 0.0592593 | 4.77778 | 3.11111 | 2.66667 |
| *O. huajuapensis* | *O. tehuacana* | *O. tehuantepecana* | 0.714286 | 0 | NA | 0.172414 | 9.72222 | 2 | 0.333333 |
| *O. huajuapensis* | *O. tehuacana* | *O. velutina* | 0.409836 | 1.43575 | 0.0755366 | 0.193798 | 3.61111 | 2.38889 | 1 |
| *O. huajuapensis* | *O. velutina* | *O. tehuantepecana* | 0.75 | 0 | NA | 0.137931 | 7 | 1.55556 | 0.222222 |
| *O. lasiacantha* | *O. streptacantha* | *O. pilifera* | 0.296 | 1.0656 | 0.143302 | 0.283525 | 52.2778 | 4.5 | 2.44444 |
| *O. pilifera* | *O. tehuacana* | *O. lasiacantha* | 0.206107 | 0.816209 | 0.20719 | 0.0206422 | 6 | 2.19444 | 1.44444 |
| ***O. lasiacantha*** | ***O. pilifera*** | ***O. tehuantepecana*** | **0.609756** | **1.70868** | **0.0437547** | **0.308642** | **10.6667** | **5.5** | **1.33333** |
| *O. velutina* | *O. pilifera* | *O. lasiacantha* | 0.179487 | 0.536875 | 0.295677 | 0.0321101 | 6.77778 | 3.83333 | 2.66667 |
| *O. lasiacantha* | *O. streptacantha* | *O. tehuacana* | 0.314286 | 1.24228 | 0.107067 | 0.424242 | 51.4167 | 4.47222 | 2.33333 |
| ***O. lasiacantha*** | ***O. streptacantha*** | ***O. tehuantepecana*** | **0.454545** | **2.78351** | **0.0026887** | **0.185185** | **60.6667** | **4** | **1.5** |
| *O. lasiacantha* | *O. streptacantha* | *O. velutina* | 0.26087 | 0.930436 | 0.176073 | 0.210526 | 53.8333 | 4.83333 | 2.83333 |
| ***O. lasiacantha*** | ***O. tehuacana*** | ***O. tehuantepecana*** | **0.891892** | **3.12162** | **0.00089929** | **0.407407** | **10.4167** | **5.83333** | **0.333333** |
| *O. velutina* | *O. tehuacana* | *O. lasiacantha* | 0.306667 | 1.08256 | 0.139502 | 0.0592784 | 6 | 4.08333 | 2.16667 |
| ***O. lasiacantha*** | ***O. velutina*** | ***O. tehuantepecana*** | **0.885714** | **2.65714** | **0.0039403** | **0.382716** | **8.5** | **5.5** | **0.333333** |
| *O. pilifera* | *O. tehuacana* | *O. streptacantha* | 0.338346 | 1.01522 | 0.155001 | 0.142857 | 3.31481 | 1.64815 | 0.814815 |
| *O. streptacantha* | *O. pilifera* | *O. tehuantepecana* | 0.384615 | 0.860026 | 0.194887 | 0.151515 | 10.2222 | 3 | 1.33333 |
| *O. velutina* | *O. pilifera* | *O. streptacantha* | 0.22449 | 0.581323 | 0.280511 | 0.0990991 | 4.22222 | 3.33333 | 2.11111 |
| *O. pilifera* | *O. tehuacana* | *O. tehuantepecana* | 0.666667 | 0 | NA | 0.142857 | 10.0556 | 1.66667 | 0.333333 |
| *O. tehuacana* | *O. pilifera* | *O. velutina* | 0.0802139 | 0.242467 | 0.404209 | 0.047619 | 3.48148 | 1.87037 | 1.59259 |
| *O. pilifera* | *O. velutina* | *O. tehuantepecana* | 0.6 | 0 | NA | 0.107143 | 8.44444 | 1.33333 | 0.333333 |
| ***O. streptacantha*** | ***O. tehuacana*** | ***O. tehuantepecana*** | **0.818182** | **2.10476** | **0.0176563** | **0.272727** | **10.0556** | **3.33333** | **0.333333** |
| *O. streptacantha* | *O. tehuacana* | *O. velutina* | 0.370787 | 1.30301 | 0.0962854 | 0.244444 | 3.61111 | 3.38889 | 1.55556 |
| *O. streptacantha* | *O. velutina* | *O. tehuantepecana* | 0.8 | 0 | NA | 0.242424 | 8 | 3 | 0.333333 |
| *O. velutina* | *O. tehuacana* | *O. tehuantepecana* | 0.333333 | 0 | NA | 0.04 | 7.16667 | 0.666667 | 0.333333 |
