# Supplementary figures and images for: Unraveling Reticulate Evolution in Opuntia (Cactaceae) From Southern Mexico
Source: Front Plant Sci. 2021 Jan 13;11:606809. doi: 10.3389/fpls.2020.606809 (PMC7838128; doi:10.3389/fpls.2020.606809)

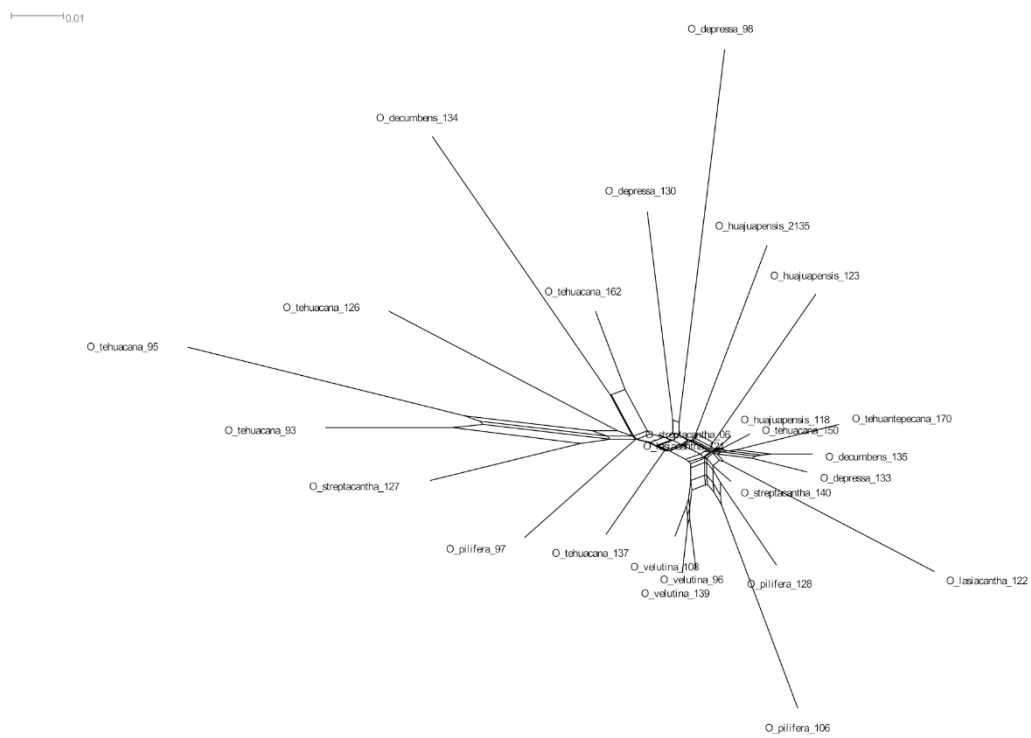

**Supplementary Figure 1.** Neighbor net for 26 *Opuntia* individuals from 9 species.

Supplement: Supplementary Figure 1 — | Neighbor net for 26 Opuntia individuals from 9 species. [file Data_Sheet_1.PDF]
